# Cohort-Based Evaluation of the Risk of Low Back Pain After Total Hip Arthroplasty: A Long-Term Study

**DOI:** 10.3390/life15020248

**Published:** 2025-02-06

**Authors:** Francisco José Gallego-Peñalver, Armando Chaure-Pardos, Silvia Beatriz Romero-de-la-Higuera, Eva María Gómez-Trullén

**Affiliations:** 1 Departmento de Fisiatría y Enfermería, iHealthy Research Group IIS Aragón, Universidad de Zaragoza, C. Domingo Miral s/n, 50009 Zaragoza, Spain; evagomez@unizar.es; 2 Rehabilitation Department, Hospital Obispo Polanco, 44002 Teruel, Spain; 3 Public Health and Immunization Surveillance Service, General Directorate of Public Health, Government of Aragón, Vía Univérsitas, 36, 50017 Zaragoza, Spain; achaure@salud.aragon.es; 4 Grupo de Investigación en Servicios Sanitarios de Aragón (GRISSA), 50009 Zaragoza, Spain; 5 Rehabilitation Department, Hospital Francesc de Borja, 46702 Gandía, Spain; silviabrh91@gmail.com

**Keywords:** hip osteoarthritis, total hip arthroplasty, low back pain, postoperative complications, long term effects, cohort analyses

## Abstract

Low back pain (LBP) is a potential complication after total hip arthroplasty (THA). However, some studies suggest that THA not only alleviates joint pain but also resolves LBP in up to 88.2% of patients. Most of these observations are limited to short-term follow-ups. This study investigates the long-term relationship between THA and LBP, challenging the notion that THA resolves LBP. A retrospective review was conducted on 236 patients who underwent THA (2010–2020). Multiple statistical models were applied, including unadjusted unmatched, adjusted unmatched, adjusted matched, and target trial emulation with 7887 subjects, to evaluate LBP incidence. Of the 236 patients, 119 developed postoperative LBP. The unadjusted unmatched analysis showed a relative risk (RR) of 2.23 (95%CI: 1.5–3.2). Adjusting for age, sex, body mass index (BMI), and recruitment period reduced the RR to 1.64 (95%CI: 1.0–2.6). The adjusted matched analysis showed an RR of 1.09 (95%CI: 0.4–3.0), while the target trial emulation simulated an RR of 1.03 (95%CI: 0.7–1.8), indicating no significant differences. Despite an apparent initial association, adjusted analyses do not support a significant long-term relationship between THA and LBP. No reduction in postoperative LBP incidence was observed, suggesting THA is safe regarding LBP risk but lacks a curative effect. Rigorous confounding adjustment is essential in retrospective studies.

## 1. Introduction

Hip osteoarthritis (HOA) is a chronic degenerative condition that causes pain, stiffness, and limited mobility in both the hip and lumbar regions, being one of the leading causes of disability in adults. The prevalence is estimated between 8% and 10.4% in the general population [1,2], with geographical variations and a marked increase with age [3,4,5]. THA has been established as the most effective surgical treatment to relieve pain and restore function in patients with advanced HOA [6]. Despite the clear benefits of THA, this surgical procedure is associated with potential complications [7]. Consequently, some patients develop postoperative low back pain (LBP), which can significantly challenge the recovery process [8].

LBP is a prevalent global health issue, with lifetime prevalence rates ranging from 49% to 81%, with an annual incidence of 15–45%. It is most common between the ages of 30 and 50 and can significantly impact daily activities and quality of life [9,10,11]. The incidence of LBP associated with untreated HOA has been reported between 21.2% and 100% [12,13]. Recent studies, such as that by Vigdorchik et al., have suggested that THA can alleviate LBP in up to 82% of patients with HOA within two years of follow-up [14]. However, there is a possibility that THA may generate biomechanical changes in the lumbar spine, accelerating disc degeneration and contributing to the appearance of new episodes of LBP in the long term, including injuries to the psoas muscle [15,16,17].

Although the biomechanical connection between the hip and lumbar spine is well documented, the factors contributing to the development of LBP after surgery continue to be the subject of study.

Given the limited evidence on the long-term impact of THA on the development of LBP, further investigation with rigorous methodology is warranted. This study hypothesizes that THA in patients with HOA will be associated with an increased risk of developing long-term LBP. The primary objective of this study is to determine the long-term impact of THA on the incidence of LBP in patients with and without pre-existing LBP, while controlling for potential confounding factors.

## 2. Materials and Methods

### 2.1. Study Design

A cohort study was conducted in patients diagnosed with HOA who underwent THA. Each patient was divided into two periods: (1) prior to THA (unexposed) and (2) after THA (exposed).

### 2.2. Participants and Follow-Up

The study included all patients who underwent THA at the University Clinical Hospital Lozano Blesa in Zaragoza between 1 January 2010 and 31 December 2020, aged between 18 and 72 years at the time of the intervention. The complete eligibility criteria are detailed in Table 1.

In the preoperative THA group, the start of follow-up was established at the time of diagnosis of HOA. Follow-up was extended until the occurrence of the first episode of LBP, the THA intervention, or 5 years, whichever occurred first. In the postoperative THA group, follow-up began on the date of the intervention and continued until the occurrence of the first episode of LBP, the end of follow-up, or 5 years, whichever occurred first.

### 2.3. Variables and Data Sources

The exposure variable was the preoperative THA or postoperative THA group. The outcome variables were the occurrence of LBP within 5 years of the start of follow-up and the time until its occurrence. To control for confounding, information on age, sex, height, and weight at the time of surgery was collected. All information was extracted from the Electronic Medical Records of the Aragon Health Service.

### 2.4. Data Analysis

Baseline characteristics were compared between groups using standardized mean differences (SMD), calculated as the difference in proportions (for categorical variables) or means (for continuous variables) divided by the pooled standard deviation.

To estimate the hazard ratios (HRs) for time to the first episode of LBP, we used Cox proportional hazards regression models. Specifically, several Cox models were fitted, all of which considered the exposure group (preoperative THA vs. postoperative THA) as the independent variable and the time to the first episode of LBP as the dependent variable. The hazard ratios (HRs) and their 95% confidence intervals were obtained by exponentiating the regression coefficients estimated in each Cox model (via the partial likelihood method). We built the following models: (i) an unadjusted model, which only included the exposure (preoperative vs. postoperative THA) as the explanatory variable; (ii) an adjusted model, which included age, sex, body mass index (BMI), and the period of follow-up initiation as covariates; (iii) a model for paired data, wherein, for each patient, we paired the preoperative THA and postoperative THA periods, adjusting for the patient’s age at the start of each follow-up period. This approach was implemented using a stratified Cox model, where each subject’s pair was treated as a stratum. The proportional hazards assumption was evaluated by examining Schoenfeld residuals, and no major deviations were identified.

### 2.5. Sensitivity Analysis

To assess the potential existence of selection or confounding biases, two additional sensitivity analyses were performed. First, the analysis was conducted with a smaller restricted sample, applying only the absolute exclusion criteria. Second, a target trial emulation study design was implemented. This type of design has been described in other articles [18]. This approach first involves specifying the protocol of a hypothetical target trial and then emulating the trial as faithfully as possible using observational data.

The selection criteria for the hypothetical trial were as follows: (1) diagnosed with HOA, (2) had not previously undergone THA prior to the start of the trial, (3) had not developed LBP between diagnosis and the start of the trial, and (4) did not meet any of the exclusion criteria in Table 1. At the start of the trial, subjects were assigned to one of two groups, namely (1) not intervened and (2) THA-intervened, based on whether they underwent the surgery during the month of trial initiation. Subsequently, subjects were followed until they developed a first episode of LBP, 5 years passed, the end of the study follow-up was reached, or, in the case of the non-intervened group, they underwent THA, whichever occurred first.

Finally, through a pooled logistic regression approach, we estimated the 5-year cumulative incidence of LBP in both the intervention and comparison groups. By comparing the predicted risk in each group, we then derived a marginal risk ratio (RR) (i.e., the ratio of the estimated incidence in the intervention group to that in the comparison group). In our case, the hypothetical trial was emulated with the available data and replicated 132 times, considering as the start date each month between 1 January 2010 and 31 December 2020. All analyses were performed using R software 4.4.1.

### 2.6. Ethical Aspects

This study was approved by the Ethics Committee for Research of the Autonomous Community of Aragon with the code C.P.-C.I.-PI21/346 and registered in the ClinicalTrial.gov database with the identifier NCT05647629. Authorization was obtained from the hospital’s medical director to conduct this study.

## 3. Results

**Figure 1:**
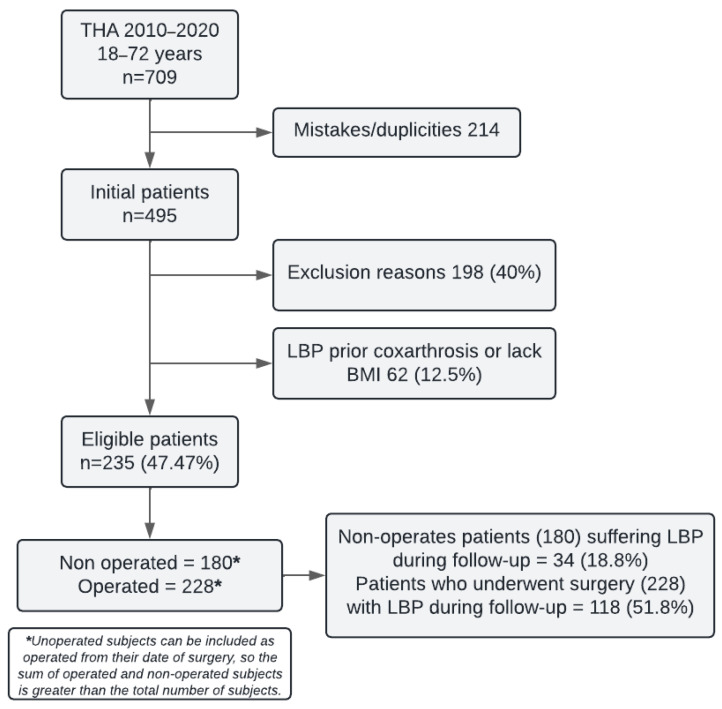
Flow chart of patients included in the study.

A total of 495 subjects diagnosed with HOA and undergoing THA were identified at the hospital during the 2010–2020 period. After applying exclusion criteria, 198 patients were excluded. Additionally, 62 more patients were excluded due to LBP prior to the diagnosis of coxarthrosis or lack of BMI data, leaving a total of 235 eligible patients for the study. A total of 180 patients were included in the preoperative THA group and 228 in the postoperative THA group. Each patient contributed data to both pre- and post-surgery periods where applicable. Of the 235 patients, 173 (73.6%) were included in both groups, while 7 (3.0%) and 55 (23.4%) were exclusive to the pre- and post-surgery groups, respectively (Figure 1).

Of the subjects included in the preoperative THA group, 34 experienced an episode of LBP during a median follow-up of 35 months (IQR: 15–72). In the postoperative THA group, 118 patients experienced an episode of LBP during a median follow-up of 51 months (IQR: 30–83). From the preoperative THA group, 173 subjects were subsequently operated on and were also included in the postoperative THA group.

### 3.1. Baseline Characteristics and Initial Follow-Up Period

The proportion of men and women is similar in both groups, with a slight difference in median age. BMI was constant between the two groups, with an average value of 29 (IQR: 26–32) (Table 2). The first records obtained date from 2001, with the last ones dating from 2022. Over this 21-year period, the greatest pre-surgical follow-up accumulated between 2006 and 2015 and the post-surgical group accumulated between 2011 and 2020 (Table 2).

### 3.2. Survival Function and HR of LBP During 5 Years of Follow-Up

**Figure 2:**
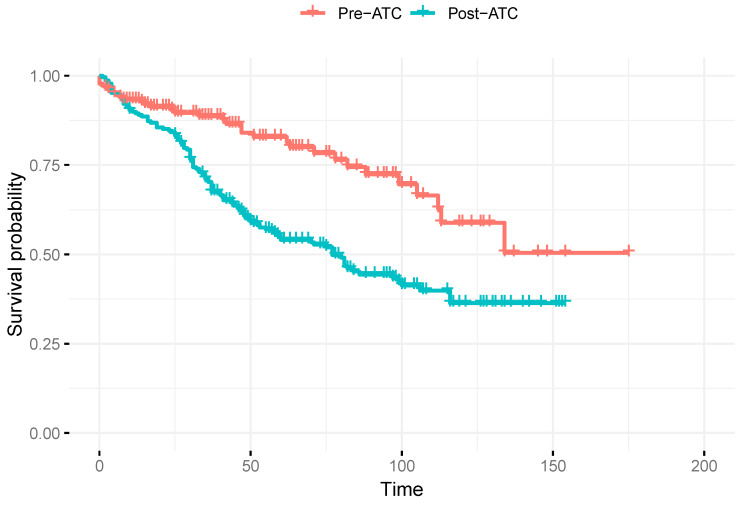
Kaplan–Meier survival curves of LBP incidence in non-intervened subjects with OA (pre-THA) compared to intervened subjects with LBP (post-THA).

Table 3 presents the HRs for the development of LBP after 5 years of follow-up across the different analysis models. The unadjusted model estimated an HR of 2.2 (95%CI: 1.5–3.2) for patients in the post-THA period compared to the preoperative THA period, with their survival function over time shown in Figure 2. However, after adjusting for age, sex, BMI, and the period of study inclusion, the HR decreased to 1.6 (95%CI: 1.0–2.6). In the matched and age-adjusted analysis, the HR was further reduced, showing a value of 1.1 (95%CI: 0.4–3.0).

### 3.3. Sensitivity Analysis Outcomes

Replicating the matched analysis and excluding only subjects who met the absolute exclusion criteria, an LBP HR of 0.8 (95%CI: 0.3–1.8) was estimated for the postoperative THA period compared to the preoperative THA period. This suggests that matching and adjusting for age can balance the baseline differences between the groups. Finally, the approach using target trial emulation, which adjusts for age, sex, BMI, disease progression time, and the period of study inclusion, estimated a relative risk (RR) of 1.04 (95%CI: 0.7–1.8).

## 4. Discussion

Previous studies have reported a prevalence of LBP of around 49.4% and a recovery rate of 88.2% after THA with a two-year follow-up [8,14]. However, this short-term follow-up period may not fully capture the prolonged impact of muscle atrophy on lumbar biomechanics, underscoring the need for further long-term investigations.

Possible causes of low back pain following total hip arthroplasty (THA) include psoas tenotomy during hip reduction, iatrogenic femoral nerve injury, intramuscular hematomas, lower limb lengthening, and muscle irritation or tendinopathy secondary to friction with the acetabulum. These events can lead to progressive psoas atrophy, contributing to the development of low back pain [19].

The initial hypothesis of this study was based on the potential long-term biomechanical alterations that may arise due to damage and weakness of the psoas muscle following THA [8,15,20,21]. This powerful, bilateral muscle plays a crucial role in lumbopelvic biomechanical control and stability. Unilateral damage could result in an asymmetric balance of tensile forces at its dorsolumbar origin (T12 to L4), leading to facet joint overload in the lumbar structures and progressive long-term damage.

Eligibility criteria were defined to ensure reliable long-term outcomes. Age was limited to 18–72 years to reduce loss to follow-up due to natural causes. Patients with previous lower extremity fractures were excluded to avoid confounding orthopedic factors, and those with significant mental impairments were excluded due to potential difficulties with follow-up. However, it is important to note that our reliance on electronic medical records (EMRs) may not capture care received outside the hospital network (e.g., private clinics), potentially leading to unmeasured data gaps. Despite this, all patients had at least one documented follow-up interaction within the system, indicating ongoing clinical engagement. These measures aimed to identify patients with OA without other comorbidities and strengthen the internal validity of the study, while acknowledging the inherent constraints of real-world EMR data.

Our sample revealed a similar distribution between men and women (64–62% and 36–38%, respectively) and an average BMI of 29, ensuring baseline comparability between groups. In the unadjusted analysis, the occurrence of postoperative LBP had a median duration of 25 months (IQR: 3–66, SD 1.35), suggesting an earlier manifestation in the surgical group, possibly due to medium-to-long-term psoas atrophy (Table 2 and Figure 2). This temporal finding indicates that the onset of LBP after THA may not be immediate but rather develops over a variable time interval [15,22], with a greater dispersion after the initial 25 months, the time required for the atrophied psoas to cause biomechanical problems in the lumbar spine.

Initially, our unadjusted analyses revealed a higher incidence of LBP in THA patients, with an HR of 2.23 (95%CI: 1.5–3.2). However, when adjusting for relevant confounding variables such as age, sex, BMI, and recruitment period, this association attenuated to an HR of 1.64 (95%CI: 1.0–2.6). Subsequently, in a matched adjusted analysis, where we compared each patient in the preoperative and post-surgical periods, the HR decreased to 1.09 (95%CI: 0.4–3.0), indicating that THA alone does not increase the risk of LBP. These results are consistent with those obtained through target trial emulation, where we calculated the 5-year RR as 1.03 (95%CI: 0.7–1.8), suggesting that there are no differences in the long-term risk of LBP between patients who underwent the intervention and those who did not.

“Target trial emulation” is an advanced epidemiological technique that aims to replicate the conditions of a randomized clinical trial using observational data [23]. This approach is particularly valuable when conducting a clinical trial is infeasible due to economic, ethical, or logistical reasons [24]. However, it is important to note that, like other observational studies, target trial emulation cannot fully control for unknown or unmeasured confounding factors, which may impact the validity of the obtained results [25].

Despite the initial findings suggesting an association between THA and LBP, a rigorous multivariable analysis revealed that this relationship is primarily due to confounding factors. The simulation of a clinical trial supported these results, indicating that surgery cannot be considered either protective or causative of subsequent LBP. This study, with its retrospective design, methodological rigour, and extensive follow-up, underscores the importance of controlling for confounding variables in the evaluation of complex data. The results obtained contribute to a better understanding of the risk profile associated with THA.

Previous investigations, such as that conducted by Vigdorchik et al., have reported postoperative improvements [14]. However, our study suggests that the risk of developing LBP is similar among patients diagnosed with HOA, regardless of whether they receive surgical treatment. Therefore, THA cannot be considered an intervention that improves LBP in addition to hip pain. These findings are consistent with research indicating that muscle atrophy may result from disuse, not only of the psoas muscle [15], but also from the weakness of other muscles involved in hip control [26,27]. Furthermore, these results tend to be consistent, regardless of the surgical technique employed [28].

Our findings support the evidence suggesting that THA does not increase the incidence of postoperative LBP, but it is also not a technique that definitively solves this problem. Therefore, it should not be promoted as a definitive solution. It is essential that patients be properly informed that surgery does not guarantee the complete disappearance of their symptoms. A multidisciplinary approach, including physical therapy, exercise, nutritional advice, and in certain cases, pharmacological treatments, may be necessary for the optimal and effective management of LBP.

While the results of this study suggest that THA does not significantly increase the long-term risk of LBP, it is important to highlight strategies that may help reduce this risk. When considering the surgeon’s role, the success of the procedure largely depends on their expertise in performing the intervention without damaging surrounding structures. Additionally, patients play a crucial role in their recovery. Postoperative rehabilitation, including strength training exercises, is essential to support the restoration of muscle function and stability around the hip and lumbar regions [29]. Lifelong maintenance of these exercises is recommended to prevent the onset of new episodes of LBP and improve long-term functional outcomes.

A critical appraisal of this study must consider its limitations, particularly the potential for confounding bias introduced by its retrospective design. Additional studies, preferably with prospective and controlled designs, are needed to confirm and expand these findings, as well as to explore more deeply the underlying factors that could influence the relationship between THA and long-term LBP.

## 5. Conclusions

This study, employing advanced epidemiological analysis techniques, found no conclusive evidence that THA increases the risk of LBP. The analysis estimated a 5-year RR of 1.03 in patients undergoing THA, indicating that the surgery neither significantly increases nor decreases the long-term risk of LBP compared to those who did not undergo surgical intervention. The initial association observed was no longer statistically significant after adjusting for key confounders such as age, sex, BMI, and disease progression time.

The results of this study refute the hypothesis that THA increases the risk of postoperative LBP, but they also contradict the idea that THA may resolve pre-existing LBP. These findings highlight the importance of considering demographic and clinical factors when evaluating the risks associated with THA, providing a more solid basis for making informed clinical decisions.

In summary, this study offers a critical perspective on the relationship between THA and the occurrence of postoperative LBP, underscoring the need for further research to improve patient quality of life and optimize clinical outcomes for those undergoing this intervention.

## Figures and Tables

**Table 1:** Inclusion criteria, partial exclusion criteria, and absolute exclusion criteria.

Inclusion Criteria	
- Age 18–72 years at the time of intervention. - THA performed between 2010 and 2020.	
Absolute Exclusion Criteria	
- Previous lower limb or axial fracture - THA due to fractures. - Lower limb amputations. - Revision THA surgeries. - Inflammatory diseases. - Neurodegenerative diseases. - Mental illness. - Patients whose Electronic Medical Record only contains the surgical report.	- Avascular necrosis of the femoral head. - Legg–Calvé–Perthes disease. - Hip dysplasias. - Congenital skeletal abnormalities. - Vertebral surgery prior to THA. - Scoliosis. - Scheuermann’s disease. - Neoplasms affecting the axial skeleton. - Bone or prosthetic infections.
Partial Exclusion Criteria	
- Bilateral total hip prosthesis. - Chronic alcoholism. - Cervical surgery.	- Previous knee arthroscopy. - Posterior knee replacement. - Post-THA leg length discrepancy.
Other Exclusion Criteria	
- Insufficient data on covariates. - Presence of low back pain prior to a diagnosis of coxarthrosis.	

**Table 2:** Baseline characteristics and standardized differences (SMD) among individuals included in the study. (BMI = body mass index; IQR = interquartile range; SMD = standardized mean difference).

Characteristics	Pre-Surgery (n = 180)	Post-Surgery (n = 228)	SMD
Sex			0.04
Male	115 (64%)	141 (62%)	
Female	65 (36%)	87 (38%)	
Median age, in years (IQR)	57 (52–63)	61 (55–67)	0.46
BMI, in Kg/cm^2^ (IQR)	29 (26–32)	29 (26–32)	0.01
Median disease progression, in months (IQR)	0 (0–0)	25 (3–66)	1.35
Follow-up start period			1.18
2001–2005	14 (7.8%)	0 (0%)	
2006–2010	64 (36%)	11 (4.8%)	
2011–2015	81 (45%)	112 (47%)	
2016–2020	21 (12%)	105 (46%)	

**Table 3:** Estimated HR and risk ratio after 5 years of follow-up in individuals included in the study.

Model	Hazard Ratio (95% CI)		Risk Ratio After 5 Years of Follow-Up (95% CI)	
	Pre-ATC (Reference)	Post-ATC	Pre-ATC (Reference)	Post-ATC
Unadjusted n = 408	1	2.23 (1.5–3.2)		
Adjusted for age, sex, BMI, and period n = 408	1	1.64 (1.0–2.6)		
Paired and adjusted for age n = 346	1	1.12 (0.4–3.1)		
Paired and adjusted for age n = 492 *	1	0.77 (0.3–1.8)		
Target trial emulation ** n = 7887 person trials			1	1.04 (0.7–1.8)

* Only absolute exclusion criteria were applied in this analysis. ** Adjusted for age, sex, BMI, disease progression, and period.

## Data Availability

The data supporting the findings of this study are available from the corresponding author upon reasonable request. Data are not publicly available due to privacy restrictions and agreements with the hospital from which they were obtained.

## Abbreviations

The following abbreviations are used in this manuscript:

HOA	Hip osteoarthritis
THA	Total hip arthroplasty
LBP	Low back pain
BMI	Body mass index
HR	Hazard ratio
